# In-Hospital Surgery as a Risk Factor for Onset of AmpC-Producing *Escherichia coli* Blood Stream Infections

**DOI:** 10.3390/diseases6030071

**Published:** 2018-08-01

**Authors:** Ruchir Chavada, Deborah Tong, Michael Maley

**Affiliations:** 1 Department of Microbiology and Infectious Diseases, NSW Health Pathology -Central Coast, Gosford, NSW 2250, Australia; 2 Pharmacy Department, Central Coast Local Health District, Gosford, NSW 2250, Australia; deborahykt@gmail.com; 3 Department of Microbiology and Infectious Diseases, NSW Health Pathology-South West Sydney, Sydney, NSW 2170, Australia; Michael.Maley@health.nsw.gov.au

**Keywords:** AmpC, risk factors, surgery, blaCMY, blood stream infections

## Abstract

There has been a progressive rise in the incidence of blood stream infections (BSI) caused by multidrug-resistant Gram-negative organisms (MDR GN), which cause increased morbidity and mortality. For this reason, recent studies have focused on risk factors of acquisition of carbapenemase-producing Enterobacteriaceae and extended-spectrum beta-lactamase producers. However, there is limited data on risk factors for BSI caused by AmpC-producing Enterobacteriaceae (AmpC EC), especially in low prevalence settings such as Australia. This study was performed to identify risk factors for acquisition of AmpC *E. coli*, using a retrospective matched case control design over a 3-year period. Patients with BSI caused by AmpC *E. coli* were matched with controls (third generation cephalosporin susceptible *E. coli*) by age and site of infection (*n* = 21). There was no significant difference in age, sex, clinical outcome, time to onset of BSI, recent antibiotic use (last 3 months), comorbidities (type 2 diabetes mellitus, renal failure) intensive care unit admission, underlying hematological condition, immunosuppressant use, APACHE II score, or any recent urological procedures (within last 3 months) between the two groups. On univariate analysis, the AmpC *E. coli* group were more likely to have had a surgical procedure in hospital and lived in a residential aged care facility. On multivariate logistic regression analysis, a recent surgical procedure was associated with the onset of AmpC *E. coli* BSI (Odd’s Ratio (OR) 4.78, *p* = 0.034). We concluded that in a relatively low prevalence setting such as Australia, AmpC *E. coli* BSI is potentially associated with surgery performed in hospital due to previous antibiotic exposure and longer hospitalization.

## 1. Introduction

In recent times, there has been a progressive rise in the incidence of blood stream infections (BSI) caused by multidrug-resistant Gram-negative organisms (MDR GN). While recent focus has been on carbapenemase-producing Enterobacteriaceae (CPE) organisms, extended-spectrum beta-lactamases (ESBL) and plasmid-mediated AmpC beta-lactamase producers are also frequently encountered in healthcare-associated BSI and are important causes of morbidity and mortality [1]. In Australia, although the prevalence of carbapenem resistance in Enterobacteriaceae is very low, incidence of BSI caused by ESBL-producing Enterobacteriaceae has increased [2]. Since only sporadic cases of CPE BSI occur, risk factors for such infections are difficult to gauge, however risk factors for infections caused by ESBL-producing Enterobacteriaceae have been studied [3]. AmpC-producing Enterobacteriaceae (AmpC EC) are another important category of MDR GN which frequently cause BSI in patients in both hospital and community [1]. Several risk factors for AmpC EC BSI have been identified, such as prolonged hospitalization, prior antibiotic use, especially fluoroquinolones, renal transplant, inappropriate empirical therapy and presence of indwelling urinary catheters and vascular access devices [4,5]. While recent manipulation (surgery) has been found as a risk factor in certain studies, it has not been consistently identified as a risk factor. As there is limited data on clinical outcomes and risk factors of BSI caused by AmpC-producing *Escherichia coli* in low prevalence settings such as Australia, this study was designed to further evaluate the risk factors and to identify whether surgery performed in hospital was associated with BSI caused by AmpC *E. coli*. 

## 2. Methods

This was a retrospective matched case control study over a 3-year period (2012–2014). The microbiology laboratory in southwestern Sydney serves 5 hospitals with a combined bed total of about 1800. There is a heterogenous case mix of patients in these hospitals, including patients who have had haemopoietic stem cell transplantation neurosurgery and cardiothoracic surgery. Patients who visited friends and families overseas and recently arrived immigrants also form a large population in the area. To design a case-control study we matched patients with BSI caused by AmpC *E. coli* (cases) to those patients who had a third-generation cephalosporin susceptible *E. coli* (controls). To minimize confounding factors that could be caused by severity of illness and comorbidities, the cases and controls were matched by age and site of infection. AmpC detection was performed by both phenotypic methods using boronic acid (inhibitor) and clavulanate (inducer) as per Clinical & Laboratory Standards Institute (CLSI) recommendations, as well as genotypic method using multiplex polymerase chain reaction (PCR) [6,7]. Clinical information was gathered from electronic medical records.

### Statistical Tests

Chi-square tests for categorical variables and t-test for continuous variables were performed. Factors associated with a *p* value of <0.1 in univariate analysis were included in multivariate logistic regression analysis (MLA) using SPSS (IBM v23.0, CA, USA). 

Ethics approval for this study was obtained from the local human research ethics committee (HREC) with the following approval number SWSLHD HREC 14/005.

## 3. Results

A total of 21 cases of AmpC *E. coli* were compared with 21 cases of susceptible *E. coli* (Table 1). There was no significant difference in age, sex, clinical outcome, time to onset of BSI, recent antibiotic use (within last 3 months), comorbidities (type 2 diabetes mellitus, renal failure) intensive care unit admission, underlying hematological condition, immunosuppressant use, Acute Physiologic Assessment and Chronic Health Evaluation (APACHE) II score, or any recent urological procedure (within last 3 months) between the two groups. On univariate analysis, the AmpC *E. coli* group were more likely to have had a surgical procedure in hospital and lived in a residential aged care facility. 7/11(63%) of the procedures performed were gastrointestinal surgeries including 2 endoscopic retrograde cholangiopancreatography (ERCP), 2 orthopedic surgeries and 1 neurosurgical shunt placement. All 7 patients were on greater than 72 h of antibiotic therapy prior to surgery. In the control group, out of 4 patients who had surgery, 2 had orthopedic surgery, 1 had biliary surgery and 1 had vascular surgery. 7/21 patients (34%) had received antibiotics for an average of 2 weeks (ceftriaxone, amoxicillin-clavulanate or cephalexin) in the past 3 months in the AmpC group. Only 3/21(15%) received antibiotics for an average of 7 days in the control group. In our study about 17/21(80%) patients who developed AmpC BSI had received an average of 7 days of antibiotics which were either ceftriaxone or piperacillin-tazobactam prior to developing an AmpC *E. coli* BSI. 

On MLA, we found that only having a recent surgical procedure was associated with the onset of AmpC *E. coli* BSI (OR 4.78 [1.12–20.31], *p* = 0.034) (Table 2). *bla*CMY was isolated in 52% (11/21) cases and nil mechanism was isolated in 7 cases, while 3 had *bla*TEM beta-lactamase (phenotypically AmpC). 

## 4. Discussion

We did not find longer hospitalization, recent admission to the intensive care unit, presence of recent urinary catheter, recent receipt of antimicrobials in the community, immunosuppression, or comorbidities such as renal failure or diabetes, to be risk factors for acquisition of AmpC *E. coli* BSI, although similar associations were observed in other studies. We postulate that these differences seen in other studies could be attributed to the prevalence of such organisms in the community, severity of illness with associated comorbidities in patients and overall antimicrobial prescribing in different hospitals [2,4,5,6].

Although few studies have identified recent surgery as a risk factor for AmpC EC BSI, this has not been consistently recognized [4,5,8,9,10]. Both recent use of antimicrobials, especially oxyimino-cephalosporins (cefotaxime, ceftazidime), and prolonged hospitalization, have been found to contribute towards acquisition of the AmpC plasmid fromother Enterobacteriaceae [11,12]. In our study, we did find prior exposure to thirdrd-generation cephalosporins or beta-lactam/beta-lactamase inhibitor combinations in patients who developed AmpC BSI. Most of these antibiotics were commenced for the index episode of infection, but were escalated to a broad-spectrum agent such as a carbapenem after susceptibility results of the AmpC *E. coli* isolate were available. Chaubey et al. found that patients who had prior treatment with oxyimino-cephalosporins had worse outcomes when these agents were used for empirical therapy for AmpC BSI [13]. We did not observe this association in our study despite patients being commenced empirically on third-generation cephalosporins, as most patients received appropriate antimicrobial therapy guided by susceptibility testing when AmpC *E. coli* was isolated from blood culture. Studies have also found that plasmid-mediated AmpC beta-lactamases code for type IV pili which increase the ability for adhesion and invasion [14]. Intestinal acquisition of the AmpC plasmid due to antimicrobial pressure may eventually increase risk of such infections due to the above factors. It is interesting that use of antibiotics for surgical prophylaxis is not a significant risk for acquisition of AmpC but prolonged prior exposure to third-generation cephalosporins or fluoroquinolones has been [15]. Rand et al. showed that although patients with BSI caused by AmpC-producing organisms had a longer median length of hospital stay, we did not find this association [16]. We think that this could be due to both case and control groups having similar comorbidities and severity of illness.

In a study from Taiwan, the authors established that there was clonal spread of CMY-2 (AmpC)-producing *Klebsiella pneumoniae* in their surgical ICU and that surgery increased the risk of this transmission [17]. While we did not identify any clusters of cases or an outbreak of AmpC EC in our intensive care units, active surveillance is important to identify and institute appropriate measures early to minimize spread of such infections. In a study from New Zealand which has a similarly low prevalence of MDR GN to Australia, isolates of AmpC-producing Enterobacteriaceae were not clonal. The authors concluded that several different clonal patterns were present in such patients from the community [18]. We could not perform typing of the isolates in our study to establish these observations. These results indicate that point source outbreaks and direct person to person transmission are not major factors leading to spread of AmpC genes [19].

Availability of protocolized infection control practices and established antimicrobial stewardship (AMS) programs would contribute to limiting the spread of multi-resistant organisms in such settings. This study highlights the importance of maintaining surveillance on patients undergoing surgery in hospitals, especially those who have prolonged length of stay. Early detection of AmpC BSI in such patients is vital for providing appropriate antimicrobial therapy. With the recent implementation of the ‘Sepsis Kills’ program in Australian hospitals, there are now guidelines and pathways available for identification and management of such patients [20]. Since AMS programs have been well established in most hospitals in Australia, targeting this group of post-surgical patients with prolonged hospital stay would help in early identification and management. In patients without a clear indication of infection, antimicrobial therapy should be stopped and for others who have an uncomplicated course, intravenous to oral switch can be recommended. We believe that such measures instituted as routine part of AMS programs could lead to a reduction in BSI caused by AmpC EC.

The limitations of our study were its retrospective nature, small sample size and screening for AmpC based on phenotypic methods only. However, all the phenotypic AmpC producers were confirmed using multiplex PCR. We elected to choose AmpC-producing *E. coli* isolates only as these were the predominant multidrug-resistant organisms in our institution. The small number size over a period of 3 years is reflective of the low prevalence. Also, as mentioned earlier, we could not do the molecular typing of the isolates. 

## 5. Conclusions

In summary, we identified that in a relatively low prevalence setting such as Australia, AmpC *E. coli* BSI is potentially associated with surgery performed in hospital, as a result of previous antibiotic exposure and longer hospitalization. Surveillance to detect MDR GN organisms including AmpC EC should be done in high-risk settings such as intensive care units. AMS initiatives could be targeted toward this group of patients with early detection, review of antibiotic therapy and timely de-escalation, which can lead to a reduction in the incidence of such infections. 

## Figures and Tables

**Table 1:** Univariate analysis of AmpC-producing BSI risk factors.

Variable	AmpC EC (*n* = 21)	Third-Generation Cephalosporin-Susceptible EC (*n* = 21)	*p* Value
Age	73.3 ± 15.6	65.5 ± 13.1	0.53
Sex (male)	13	13	
Mean length of stay (IQR)	28 (3–187)	23 (4–89)	0.30
Mean duration of definitive antimicrobial therapy (days)	14.3	10.4	0.08
Onset			0.51
Hospital	10	12	
Community	11	9	
Infection type			0.19
Urinary tract infection	10	10	
Intraabdominal infection	6	7	
Others	5	4	
Recent antibiotic exposure	7	3	0.14
Surgery in hospital	11	4	0.024 *
Intensive care unit admission	9	5	0.19
Residence in aged care facility	4	0	0.035 *
Immunosuppression including chemotherapy	0	3	0.07
Urological procedure	3	3	0.98
Type 2 diabetes mellitus	3	5	0.42
Renal failure	0	1	0.31
Mean APACHE2 score	18.5 ± 3.7	17.8 ± 3.6	0.043 *
Outcome	4	6	0.46

* *p* values included in the MLA.

**Table 2:** Multivariable analysis.

Variable	Odds Ratio	95% CI		*p* Value
Surgery in hospital	4.78	1.12	20.31	0.034

## Availability of Data and Material

Since data submission to repository was not included in the ethics application, dataset is not publicly provided. However, deidentified data could be made available if required after approval from HREC committee.

## Declarations

Ethics approval and consent to participate—Ethics approval for this study was obtained from South Western Sydney Human Research Ethics Committee.
